# Non-Small Cell Lung Carcinoma Biomarker Testing: The Pathologist’s Perspective

**DOI:** 10.3389/fonc.2014.00182

**Published:** 2014-07-16

**Authors:** Elisa Brega, Guilherme Brandao

**Affiliations:** ^1^Department of Pathology, Sir Mortimer B. Davis-Jewish General Hospital, McGill University, Montreal, QC, Canada

**Keywords:** ALK, EGFR, NSCLC, adenocarcinoma, biomarker, histology, lung

## Abstract

Biomarker testing has become standard of care for patients diagnosed with non-small cell lung carcinoma (NSCLC). Although, it can be successfully performed in circulating tumor cells, at present, the vast majority of investigations are carried out using direct tumor sampling, either through aspiration methods, which render most often isolated cells, or tissue sampling, that could range from minute biopsies to large resections. Consequently, pathologists play a central role in this process. Recent evidence suggests that refining NSCLC diagnosis might be clinically significant, particularly in cases of lung adenocarcinomas (ADC), which in turn, has prompted a new proposal for the histologic classification of such pulmonary neoplasms. These changes, in conjunction with the mandatory incorporation of biomarker testing in routine NSCLC tissue processing, have directly affected the pathologist’s role in lung cancer work-up. This new role pathologists must play is complex and demanding, and requires a close interaction with surgeons, oncologists, radiologists, and molecular pathologists. Pathologists often find themselves as the central figure in the coordination of a process, that involves assuring that the tumor samples are properly fixed, but without disruption of the DNA structure, obtaining the proper diagnosis with a minimum of tissue waste, providing pre-analytical evaluation of tumor samples selected for biomarker testing, which includes assessment of the proportion of tumor to normal tissues, as well as cell viability, and assuring that this entire process happens in a timely fashion. Therefore, it is part of the pathologist’s responsibilities to assure that the samples received in their laboratories, be processed in a manner that allows for optimal biomarker testing. This article goal is to discuss the essential role pathologists must play in NSCLC biomarker testing, as well as to provide a summarized review of the main NSCLC biomarkers of clinical interest.

## Introduction

In Canada, lung cancer represents the second most common cancer in both males and females (14 and 13%, respectively), and it is the leading cause of cancer death for both sexes (1). In fact, lung cancer, with 27.2 and 26.3% mortality rate in males and females, respectively, is responsible for more deaths among Canadians than the other two leading organ-specific cancers combined [colorectal (12.7%) and prostate (10.0%) in males, and breast (13.9%) and colorectal (11,6%) in females] (1). In the United States, approximately 84% of new lung cancer cases are classified as non-small cell lung carcinomas (NSCLC), and 15% as small cell carcinomas (SCC) (2), with the majority of patients being diagnosed at advanced-stage (56%) (3). The prognosis is poor, with the overall 5-year survival rate of 6.1% for SCC and 17.1% for NSCLC (2).

Implementation of personalized targeted therapies has become a reality for a group of lung cancer patients, but this therapeutic option is usually reserved for those patients whom tumor samples have been screened for specific biomarkers. A multitude of potentially useful biomarkers have recently emerged and this list continues to grow. It has become increasingly difficult for pathologists and oncologists to define which biomarkers should be routinely tested. An expert panel in pathology and oncology, assembled by the College of American Pathologists (CAP) with representatives from the International Association for the Study of Lung Cancer (IASLC) and Association for Molecular Pathology (AMP), has recently met in an attempt to address questions regarding biomarker testing in lung cancer. The conclusions have been published in the format of testing guidelines, which presently recommends investigations of abnormalities involving only two genes: the epidermal growth factor receptor (*EGFR*) and the anaplastic lymphoma kinase (*ALK*) (4).

This review will focus on the role of the pathologist as an essential figure in the NSCLC biomarker testing process.

## Tissue/Cytological Diagnosis and Biomarker Testing

**Figure 1:**
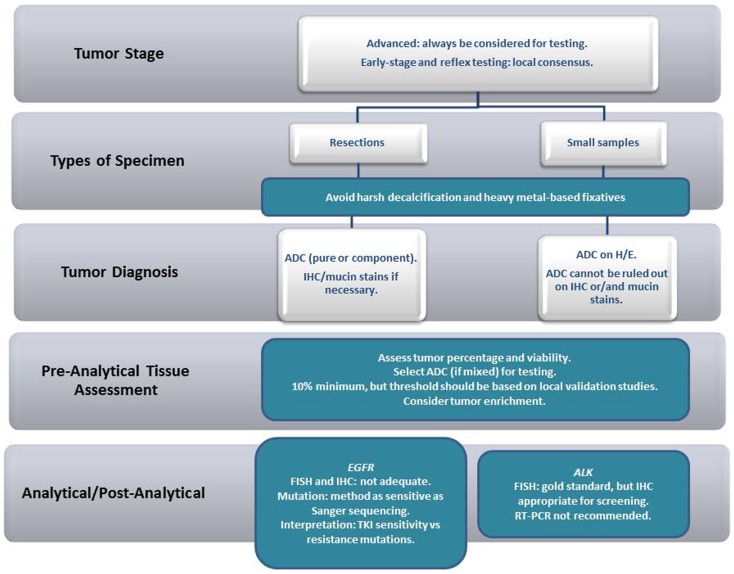
**Recommendations on specimen handling in NSCLC biomarker testing**.

NSCLC, as a standing alone diagnosis, in either tissue or cytological samples, should be avoided whenever possible. In some situations (when the tumor sample is restricted to a smear from a bronchial brushing of a poorly differentiated carcinoma, for example), further characterization might be impossible. However, in our experience, further characterization, particularly with the help of special histochemical stains for the detection of mucin (often with the use of PAS-D or mucicarmin), and/or immunocytochemistry, can be achieved in the majority of cases. From a practical point of view, samples containing adenocarcinoma (ADC) either pure or mixed should undergo biomarker testing. In small samples, the recommendations are less stringent, and, as long as an ADC component cannot be excluded, the tissue should undergo biomarker testing (irrespective of the main tumor component identified). In resections, however, when the pathologist has an opportunity to examine the lesion in its entirety, “pure” tumors [large cell carcinoma (LGC), squamous cell carcinoma (SqCC) or others] should not be tested (Figure 1).

Numerous immunomarkers are available in order to help in the sub classification of NSCLC. The most commonly used are TTF-1, Napsin-A, p63, CK 5/6, and p40 (5–13). Although, it is true that in most cases the pathologist will be reasonably at ease to sub classify NSCLC’s, in some cases, sub classification might be rather difficult. It is our understanding that if the pathologist is uncertain about the specific sub classification, then the sample should be submitted for biomarker testing.

Despite the emphasis placed on focusing on ADC for biomarker testing, it is important to highlight that there are, however, isolated reports in the literature of the detection of either *EGFR* mutations or *ALK* rearrangements in tumors classified as SqCC (14–17).

Interestingly, some genetic aberrations can be generally associated with specific NSCLC subtypes and/or clinical profile (i.e., smokers versus non-smokers) (18). ADC is the predominant histologic type associated with both *EGFR-*mutated, as well as in *ALK*-rearranged cases. However, *EGFR* mutations are particularly prevalent in those cases containing non-mucinous bronchioloalveolar (lepidic) pattern (19), while in *ALK-*rearranged ADC, the most striking correlation is made with the presence of a signet-ring component (Table 1) (20, 21).

**Table 1:** **Summary of the clinical characteristics, common genetic abnormalities and respective targeting agents of the main NSCLC biomarkers**.

Biomarkers	Gender and age	Prevalence	Tobacco	Ethnicity	ADC versus SqCC/distinctive histologic characteristics	Clinically relevant genetic abnormality	Examples of targeting agent (available or in development)
*EGFR*	Female, Younger	10-40%	Non-smokers	Asian	ADC/Non-mucinous bronchioloalveolar (lepidic)	Mutation (various, most common in-frame deletions of exon 19 and a point mutation (CTG to CGG) in exon 21)	Gefitinib, Erlotinib, Afatinib, Dacomitinib, Neratinib
*ALK*	Younger	2-6%	Non-smokers	Not distinctive	ADC/solid pattern, signet-ring cells	Translocation, inversion (*EML4-ALK* most common)	Crizotinib, LDK378
*HER2/ERBB2*	Female	1-4%	Non-smokers	Asians	ADC	In-frame insertions in exon 20	Trastuzumab Pertuzumab, Lapatinib
*ROS1*	Female, younger	0,5-2%	Non-smokers	Und.	ADC	Translocation *(ROS1-FIG)*	Crizotinib
*RET*	Younger	1-2%	Non-smokers	Not distinctive	ADC/Adenosquamous	*KIF5B–RET* and *CCDC6–RET* fusion genes	Vandetanib Cabozantinib
*KRAS*	Not distinctive	15-30%	Smokers	Caucasian	ADC/mucinous, particularly with lepidic (bronchioloalveolar) pattern	Mutations in codon 12 (majority) and 13	Selumetinib (via inhibition of MEK)
*BRAF*	Not distinctive	3% (ADC’s)	Smokers	Not distinctive	ADC	Mutations in, V600E(50%), G469A(39%), D594G(11%)	Dabrafenib, Vemurafenib, XL281, Selumetinib
*NRAS*	Und.	0.5-1%	Smokers	Und.	ADC	Mutations in codon Q61 in exon 3 (80%) and G12 (exon 2)	Selumetinib Trametinib
*FGFR1*	Not distinctive	22% of SqCC	Smokers	Not distinctive	SqCC	Amplification	PD173074
*PTEN*	Not distinctive	4-8%	Smokers	Not distinctive	SqCC	Various mutations in exon 5-8	GSK2636771
*DDR2*	Und.	2.5-3.8%	Und.	Und.	SqCC	Missense mutations, several	Imatini, Dasatinib
*MAP2K1/MEK1*	Und.	1%	Unclear	Und.	ADC	Mutations in Q56P, K57N and D67N	AZD6244, Pimasertib, Refametinib, others
*PIK3CA*	Not distinctive	2-4%	Mixed reports	Not distinctive	ADC and SqCC	Mutations in E545K AND H1047R (most common), also E542K and H1047L	Everolimus, Tensirolimus, GDC-0941, XL-147, Others
*AKT1*	Und.	1%	Und.	Und.	ADC and SqCC	Mutation in E17K	MK-2206
*MET*	Not distinctive	1-5%	Not distinctive	Und.	ADC	Amplification, protein overexpression and mutation	Vandetanib, Cabozantinib

*Und: undetermined*.

An important aspect that affects biomarker testing is the amount of available tumor present in a determined sample. This is a rather difficult topic to address, since the test sensitivity varies significantly according to the employed technique, particularly when searching for *EGFR* mutations, where normal DNA might interfere with test sensitivity (22). Nevertheless, the pathologist should provide an estimation of the percentage of tumor present in the sample, as well as, the viability of the tumor cells. It is recommended that testing sensitivity, as well as determination of limiting factors that might influence optimal results (fixative choice for example), should be defined locally, through proper validation methods. Of note, samples collected from aspiration biopsy methods, including direct lesion sampling (transbronchial needle aspiration biopsies), as well as the drainage of effusions, should be considered for biomarker testing (23–25).

## Epidermal Growth Factor Receptor

Epidermal growth factor receptor (also known as *HER-1 or Erb1*) is a member of the ErbB receptor tyrosine kinase family, which also includes *HER-2/neu (ErbB2), HER-3 (ErbB3)*, and *HER-4 (ErbB4)*. *EGFR* activation is associated with cancer cell growth, invasion, proliferation, apoptosis, tumor angiogenesis, and metastatic spread. Therefore, it plays an important role in carcinogenesis and tumor progression by activation mechanisms, including overexpression, mutation, and autocrine ligand production. These actions are accomplished through activation of the RAS/RAF/MEK/MAPK and the PI3K/AKT/mTOR pathways (26).

The two most common *EGFR* activating mutations that confer sensitivity to tyrosine kinase inhibitors (TKI) are short in-frame deletions of exon 19, and a point mutation (CTG to CGG) in exon 21 at nucleotide 2573, that results in substitution of leucine by arginine at codon 858 (L858R) (27). Despite the fact that these two mutations might represent approximately 90% of all known clinically significant *EGFR* mutation*s*, the consensus recommendations are that all *EGFR* mutations that account for at least 1% should be tested (4). It is important to emphasize that among the tested mutations, exon 20 T790M, as well as most exon 20 insertions are associated with resistance to first-generation TKI’s (28).

Epidermal growth factor receptor mutations occur at a higher frequency in tumors from East Asians than from non-Asians (30 versus 8%), from women than from men (59 versus 26%), from never smokers than from ever smokers (66 versus 22%), and in ADC’s compared with other NSCLC histologies (49 versus 2%) (29). In the United States, it is estimated that activating *EGFR* mutations are found in 15% of patients with primary lung ADC (Table 1) (30).

Turn-around-time (TAT) might be a very important factor for advanced-stage patients, whom might benefit from early institution of targeted therapy. The consensus recommends a maximum of 10 working days as an acceptable TAT from the date the laboratory receives the sample to be tested (4).

## Anaplastic Lymphoma Kinase

Translocations involving *ALK* have previously been identified in anaplastic large cell lymphomas (ALCL), and in a rare mesenchymal neoplasm known as inflammatory myofibroblastic tumor or inflammatory pseudotumor (31, 32). In lung carcinomas, *ALK* rearrangement was first demonstrated in 2007 by Soda et al. (33) when *ALK* fusion transcripts were found in 6.7% (5 out of 75) of NSCLC samples. However, the prevalence of *ALK* rearrangement in lung carcinomas varies significantly (34–36).

*ALK* rearrangements tend to be mutually exclusive with other known driver mutations in NSCLC (18). However, it has rarely been described together with *EGFR* and *PI3K* mutations (36–38).

*ALK*-rearranged NSCLC patients, when compared to *ALK*-non-rearranged, are more frequently non- or light-smokers, younger, and present with advanced clinical stage. Histologically, the tumors demonstrate most frequently ADC with solid pattern and signet-ring cells (20, 21, 39).

Although fluorescence *in situ* hybridization (FISH) is currently the gold standard method for detecting *ALK* rearrangements according to the United States Food and Drug Administration (FDA) (40), the CAP consensus accepts that, if carefully validated, immunohistochemistry can be considered as a screening method (4). This proposition is in concert with the literature, which has shown in several different articles that immunohistochemistry can be very effective in the detection of *ALK* rearrangement in lung carcinomas (41–43).

## Other Biomarkers

Currently, in over 50% of NSCLC’s, a driver oncogene can be identified (18). In addition to the previously discussed *ALK* and *EGFR* genes, several other potential targets have been uncovered in NSCLC’s, including the V-Ki-ras2 Kirsten rat sarcoma viral oncogene homolog (*KRAS*), the human epidermal growth factor receptor 2 (*HER2*), reactive oxygen species 1 (*ROS1*), v-raf murine sarcoma viral oncogene homolog B1 (*BRAF*), phosphoinositide-3-kinase catalytic alpha polypeptide (PIK3CA), c-mesenchymal-epithelial transition mitogen (*c-MET*), activated protein kinase (*MAP2K1*), fibroblast growth factor receptor (FGFR), discoidin domain receptor 2 (DDR2), phosphatase and tensin homolog (PTEN), protein kinase B (AKT), rearranged during transfection (RET), and the neuroblastoma RAS viral oncogene homolog (*NRAS)*. It is beyond the scope of this review to discuss each in detail. Current general knowledge of the characteristics of lung cancers carrying abnormalities in these genes has been summarized in Table 1 (18, 20, 21, 28, 31, 33, 36, 37, 41, 44–72).

In conclusion, targeted therapy is already a reality for many patients and it is certain that several other components will soon follow to become valid options in the therapeutic arsenal of oncologists. In view of the overwhelming amount of information being constantly generated into the molecular derangements associated with the development of lung cancer, it is not farfetched to expect that the current consensus guidelines will soon become obsolete. As a pathologist, I witness on a daily basis a continuous and inexorable change in our practice: our job no longer ends with the histological diagnosis. In fact, molecular profiling has become an integral part of the surgical pathology report. It is crucial that us pathologists embrace this new format of oncologic surgical pathology practice, and question ourselves, after each new malignant diagnosis: “what should I do now that might translate into a potential treatment alternative for this patient?”

## Conflict of Interest Statement

The authors declare that the research was conducted in the absence of any commercial or financial relationships that could be construed as a potential conflict of interest.

## Abbreviations

NSCLC, non-small cell lung carcinoma; SCC, small cell carcinomas; *EGFR*, epidermal growth factor receptor; *ALK*, anaplastic lymphoma kinase; ADC, adenocarcinoma; LGC, large cell carcinoma; SqCC, squamous cell carcinoma; TKI, tyrosine kinase inhibitors; TAT, turn-around-time; ALCL, anaplastic large cell lymphomas; FISH, fluorescence *in situ* hybridization.
